# Alkali Metal Cations Impact the Selectivity of Radical‐Mediated Electrochemical C─H Chlorination

**DOI:** 10.1002/anie.202509115

**Published:** 2025-07-23

**Authors:** Bo Wu, Ruihu Lu, Tenghui Yuan, Beijing Cai, Bingqing Wang, Bote Zhao, Shibo Xi, Ziyun Wang, Yanwei Lum

**Affiliations:** ^1^ Department of Chemical and Biomolecular Engineering National University of Singapore Singapore 117580 Republic of Singapore; ^2^ Institute of Materials Research and Engineering (IMRE), Agency for Science, Technology and Research (A*STAR), 2 Fusionopolis Way Innovis #08‐03 Singapore 138634 Republic of Singapore; ^3^ School of Chemical Sciences The University of Auckland Auckland 1142 New Zealand; ^4^ School of Environment and Energy South China University of Technology Guangzhou 510006 China; ^5^ Institute of Sustainability for Chemical, Energy and Environment (ISCE2) Agency for Science, Technology and Research (A*STAR) 1 Pesek Road 627833 Republic of Singapore

**Keywords:** Cations, Chlorination, Electrochemistry

## Abstract

Electrochemistry offers a promising route toward facilitating organic transformation reactions in a sustainable manner. However, there are often a multitude of factors at play; hence, it can be unclear how operating conditions can be rationally tuned to optimize selectivity. Here, we demonstrate how the identity of alkali metal cations in the electrolyte can control the selectivity of electrochemical C─H chlorination. Specifically, we obtained a 90.3% Faradaic efficiency with KCl as compared to 78.4% with LiCl for the conversion of cyclohexane to chlorocyclohexane at 1000 mA using an IrO*_x_* electrode. Electron paramagnetic resonance spectroscopy experiments indicate a greater propensity for Cl^−^ oxidation to generate Cl radicals in the order: K^+^ > Na^+^ > Li^+^. This leads to an increase in the selectivity toward the chlorination of cyclohexane and a concomitant decrease in competitive Cl_2_ formation. Density functional theory calculations and in situ Raman spectroscopy experiments indicate that this is likely due to a decrease in *Cl binding energy on IrO*_x_* in the presence of K^+^. These findings highlight the important role of alkali metal cations, which can be a key consideration for designing electrochemical organic synthesis systems.

## Graphical abstract

In this work, we found that alkali metal cations in the electrolyte can impact the *Cl binding energy, which controls selectivity between radical‐mediated electrochemical C─H chlorination and competing Cl_2_ formation.

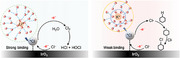

Employing electrochemistry for performing organic transformations offers an attractive route toward sustainable chemicals manufacturing.^[^ ^1^, ^2^, ^3^ ^]^ This is because electrons are used for facilitating the reactions as opposed to traditional methods that rely on stoichiometric chemical redox reagents, which can generate large amounts of waste.^[^ ^4^, ^5^, ^6^ ^]^ Furthermore, electrochemical processes^[^ ^7^, ^8^, ^9^ ^]^ can be directly powered using renewable electricity and hence has the potential to reduce the carbon intensity of chemicals manufacturing.^[^ ^10^, ^11^ ^]^ For such technology to be practical, there is a need to develop systems that can operate at reduced operating voltages, larger current densities, and improved product selectivity.

For this purpose, much of the literature has been focused on optimizing operating conditions such as the electrode material,^[^ ^12^, ^13^, ^14^, ^15^ ^]^ type of solvent,^[^ ^16^, ^17^, ^18^ ^]^ and operating temperature.^[^ ^19^, ^20^ ^]^ Despite a vast body of work in the literature on electrochemical organic synthesis, we note that there has not yet been a systematic study on the effect of alkali metal cations in the electrolyte. This is surprising since the “cation effect” has been extensively studied in many conventional electrocatalytic reactions^[^ ^21^ ^]^ such as the hydrogen evolution reaction (HER),^[^ ^22^, ^23^, ^24^ ^]^ oxygen evolution reaction (OER),^[^ ^25^, ^26^, ^27^ ^]^ and carbon dioxide reduction (CO_2_R).^[^ ^28^, ^29^, ^30^ ^]^ For instance, Bender et al.^[^ ^31^ ^]^ reported that the HER activity on Pt decreases with a larger cation size according to: Li^+^ > Na^+^ > K^+^. On the other hand, Singh et al.^[^ ^32^ ^]^ reported that the CO_2_R selectivity on Cu and Ag is higher with a larger cation size: K^+^ > Na^+^ > Li^+^.

In this work, we were motivated to study the cation effect on electrochemical organic transformations. For this purpose, we selected a simple reaction involving the electrochemical C─H chlorination of cyclohexane to chlorocyclohexane on an IrO*_x_* electrode. Based on our prior work,^[^ ^33^ ^]^ this reaction takes place through a radical mediated nonchain mechanism involving electrogenerated Cl radicals as depicted in Figure 1. Here, we found that the Faradaic efficiency (FE) and partial current density to chlorocyclohexane trends according to K^+^ > Na^+^ > Li^+^. Using electron paramagnetic resonance (EPR) spectroscopy with TEMPO radical trapping, we provide evidence that this is because larger cations promote the generation of Cl radicals and suppress competitive Cl_2_ formation. Through density functional theory (DFT) calculations and in situ Raman spectroscopy experiments, we find that this could be due to a decrease in *Cl binding energy on IrO*_x_* in the presence of larger cations in the electrolyte.

**Figure 1:**
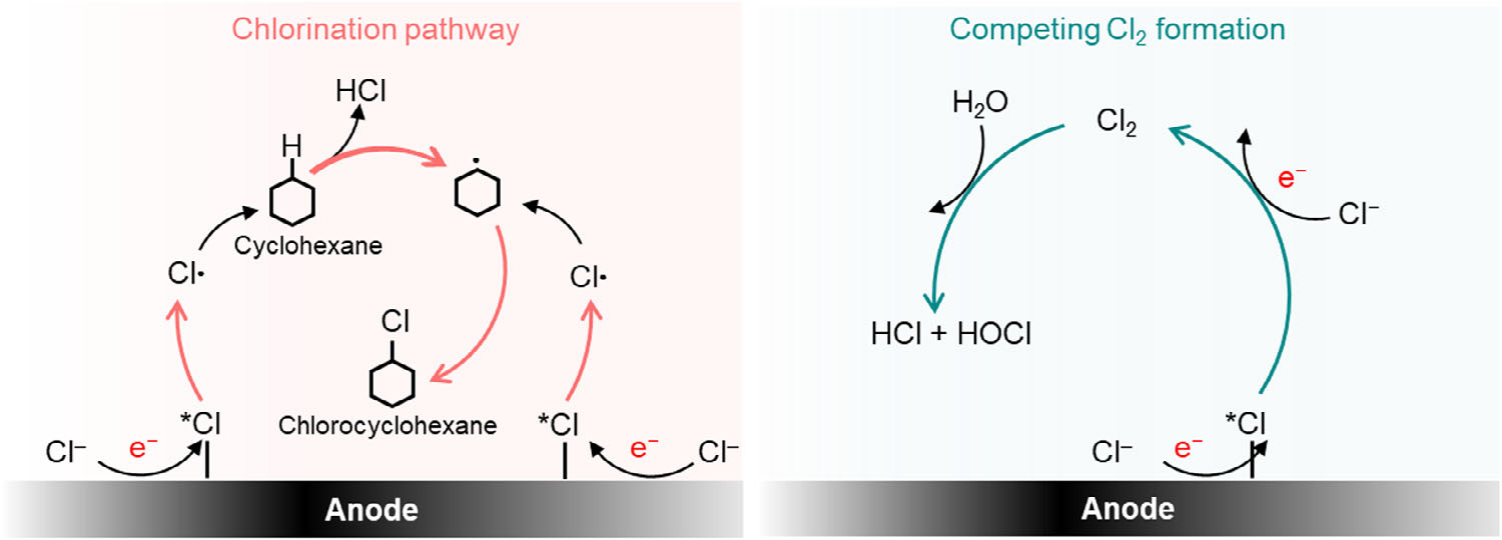
Reaction pathway schematics. Illustration of the mechanism for cyclohexane chlorination to chlorocyclohexane (left) and the competing Cl_2_ formation mechanism (right).

The electrochemical C─H chlorination of cyclohexane is performed using the same methodology as our previous work. An IrO*_x_* electrode supported on Ti mesh (Figures S1–S3) was synthesized and used as the working electrode (anode) with a geometric area of 5 cm^2^. These experiments were performed in a conventional H‐type cell, using 45 ml of electrolyte together with 5 ml of cyclohexane in the anode chamber. Here, the electrolyte consists of 1 M XCl in H_2_O, where “X” refers to the identity of alkali metal cation (Li, Na, or K). In all cases, 0.5 M H_2_SO_4_ is added as the supporting electrolyte. Due to the low solubility of cyclohexane in water, vigorous mechanical stirring is used to maintain an “emulsion”‐like condition, which is necessary to facilitate chlorination. More details on the experimental protocols employed are available in the Supporting Information methods section (Figures S4 and S5).

With this configuration, we began with linear sweep voltammetry (LSV) experiments using different alkali metal cations in the electrolyte. From the results (Figure 2a), we find that the current density increases with larger cation size: Li^+^ < Na^+^ < K^+^. This same trend was observed without addition of 0.5 M H_2_SO_4_ as the supporting electrolyte (Figure S6). We also found similar Tafel slopes for all cases, with values of 69.6 mV dec^−1^ for Li^+^, 67.3 mV dec^−1^ for Na^+^, and 57.6 mV dec^−1^ for K^+^ (Figure 2b). Electrochemical impedance spectroscopy (EIS) was also performed at a potential of 1.2 V versus Ag/AgCl for all cases and the results (Figure 2c) show that charge transfer resistance values are indeed in the order Li^+ ^> Na^+^ > K^+^. Hence, these results indicate that cations have a considerable influence on the electrochemical response, which could in turn impact the chlorocyclohexane product selectivity.

**Figure 2:**
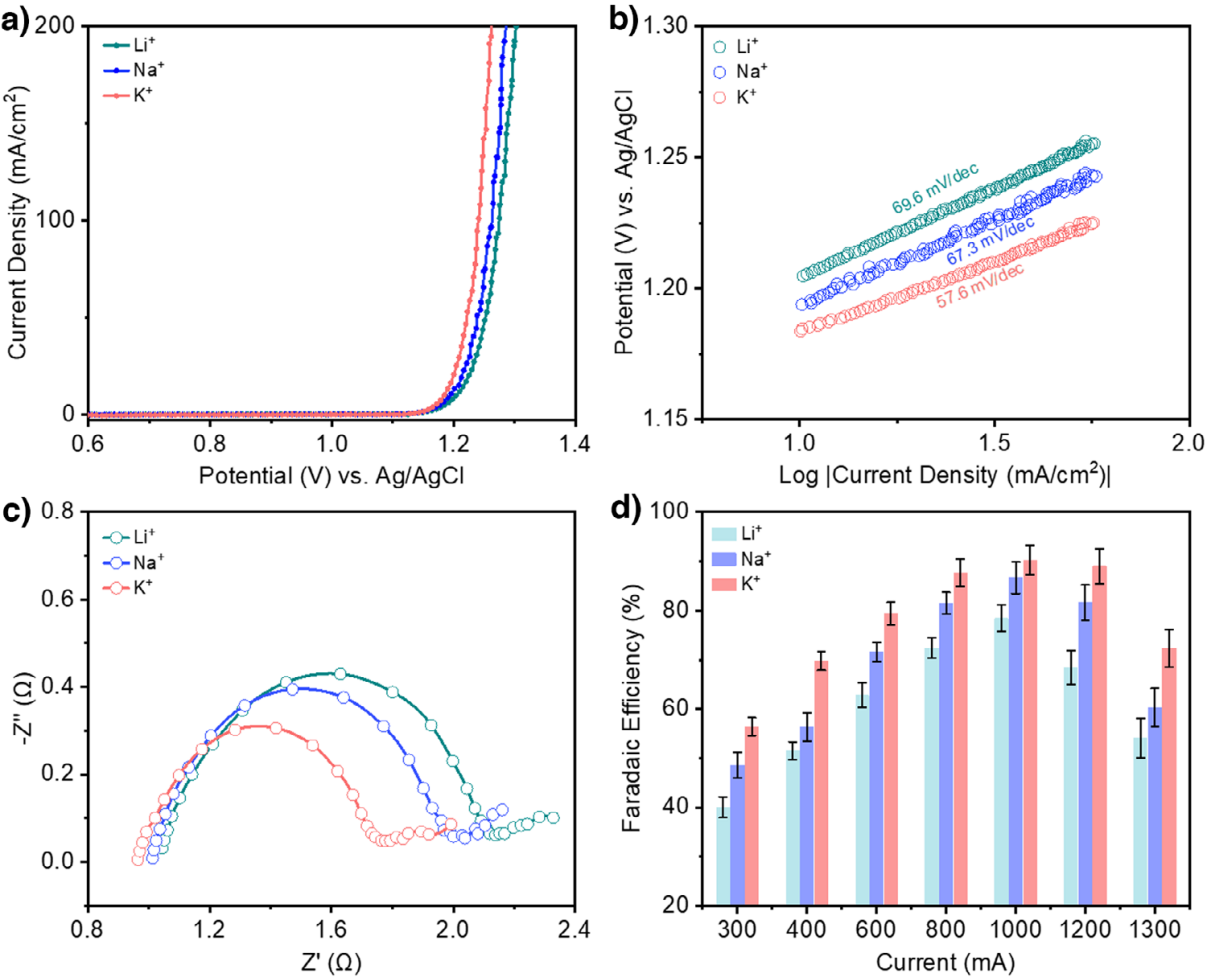
Electrochemical chlorocyclohexane production with different alkali metal cations in the electrolyte. a) LSV curves with 85% IR (voltage drop) correction with various 1 M XCl + 0.5 M H_2_SO_4_ electrolytes, where X represents either Li, Na, or K. 5 ml of cyclohexane was added to 45 ml of electrolyte and rapidly stirred to maintain an “emulsion”‐like state. b) Calculated Tafel slope values with the various alkali metal cation electrolytes employed. c) EIS data collected in the various alkali metal cation electrolytes at 1.2 V versus Ag/AgCl. d) Chlorocyclohexane FE at different applied currents for the various cation electrolytes. Note: Quantification was based on GCMS analysis and the geometric area of electrode used in all cases was 5 cm^2^. The error bars correspond to the standard deviation of at least three independent measurements, and the center value for the error bars is the average of the three independent measurements.

We proceeded to carry out constant potential electrolysis to determine the influence of the alkali metal cation on the product FE. For these experiments, we performed electrolysis at constant current values of 300, 400, 600, 800, 1000, 1200, and 1300 mA, using the same 1 M XCl + 0.5 M H_2_SO_4_ electrolytes (Table S1). At the end of each experiment, the top organic layer consisting of unreacted cyclohexane and chlorocyclohexane is removed using a separating funnel for product analysis and quantification. Fourier‐transform infrared spectroscopy (FTIR) results show there is a clear peak that corresponds to the C─Cl bond of chlorocyclohexane for all tested conditions (Figures S7–S11). Gas chromatography mass spectrometry (GCMS) analysis also confirms that chlorocyclohexane is indeed the product generated from our experiments (Figures S12 and S13).

The chlorocyclohexane product FE was also calculated based on the GCMS analysis (Figures S7–S11). The results show that the FE also follows the trend according to: K^+^ > Na^+^ > Li^+^ across the entire tested current range. For instance, a FE of 56.4% was achieved in electrolyte containing K^+^ as compared to a FE of 48.5% with Na^+^ and 40% with Li^+^ at a current of 300 mA. Similarly, at a higher current of 1000 mA, the FE was highest at 90.3% with K^+^, 86.7% with Na^+^, and 78.4% with Li^+^ (Figure 2d). However, at the higher currents, the FE was observed to decrease, with values of 72.4%, 60.3%, and 54.1% for K^+^, Na^+^ and Li^+^, respectively at a current of 1300 mA. We note that there is always a non‐negligible amount of missing FE (>10%) in all cases. To account for this, we performed iodometric titration (Figure S14), which allows us to detect and quantify any unreacted chlorine and hypochlorite species remaining in the aqueous electrolyte (Tables S2–S4). From this, we found that the formation of such species tends to be more prevalent in the Li^+^ electrolyte. Hence, these results indicate that larger cations favor the formation of chlorocyclohexane over Cl_2_ evolution, which we postulate to be due to a greater propensity for Cl radical formation.

To explore this hypothesis, we employed EPR spectroscopy and TEMPO free radical trapping with different alkali metal cations in the electrolyte. Based on our prior work,^[^ ^33^ ^]^ we showed that electrogenerated Cl radicals can be trapped by TEMPO to form TEMPO‐Cl. Hence, a more rapid decay of the TEMPO peak area in EPR spectroscopy would be indicative of a higher propensity for Cl radical generation. For these experiments, we used a TEMPO concentration of 0.1 mM with the same 1 M XCl + 0.5 M H_2_SO_4_ electrolytes. An anodic current density of 100 mA cm^−2^ was applied for the tests, which was paused at regular intervals for EPR spectroscopy measurements. For all electrolytes, we found similar TEMPO peak intensity values under open‐circuit voltage conditions (time = 0 s). Upon application of the anodic current, we started to observe a gradual decay of the TEMPO signals with time (Figure 3a–c), which eventually disappears in all cases. By comparing the TEMPO intensity at different electrolysis times, we observed that the signal decays the fastest with K^+^ and the slowest with Li^+^ (Figure 3d). Specifically, the TEMPO intensity goes to near zero after 15 s for K^+^, 25 s for Na^+^, and 40 s for Li^+^. Importantly, these EPR results indicate that K^+^ has the highest propensity for Cl radical formation, which supports our working hypothesis.

**Figure 3:**
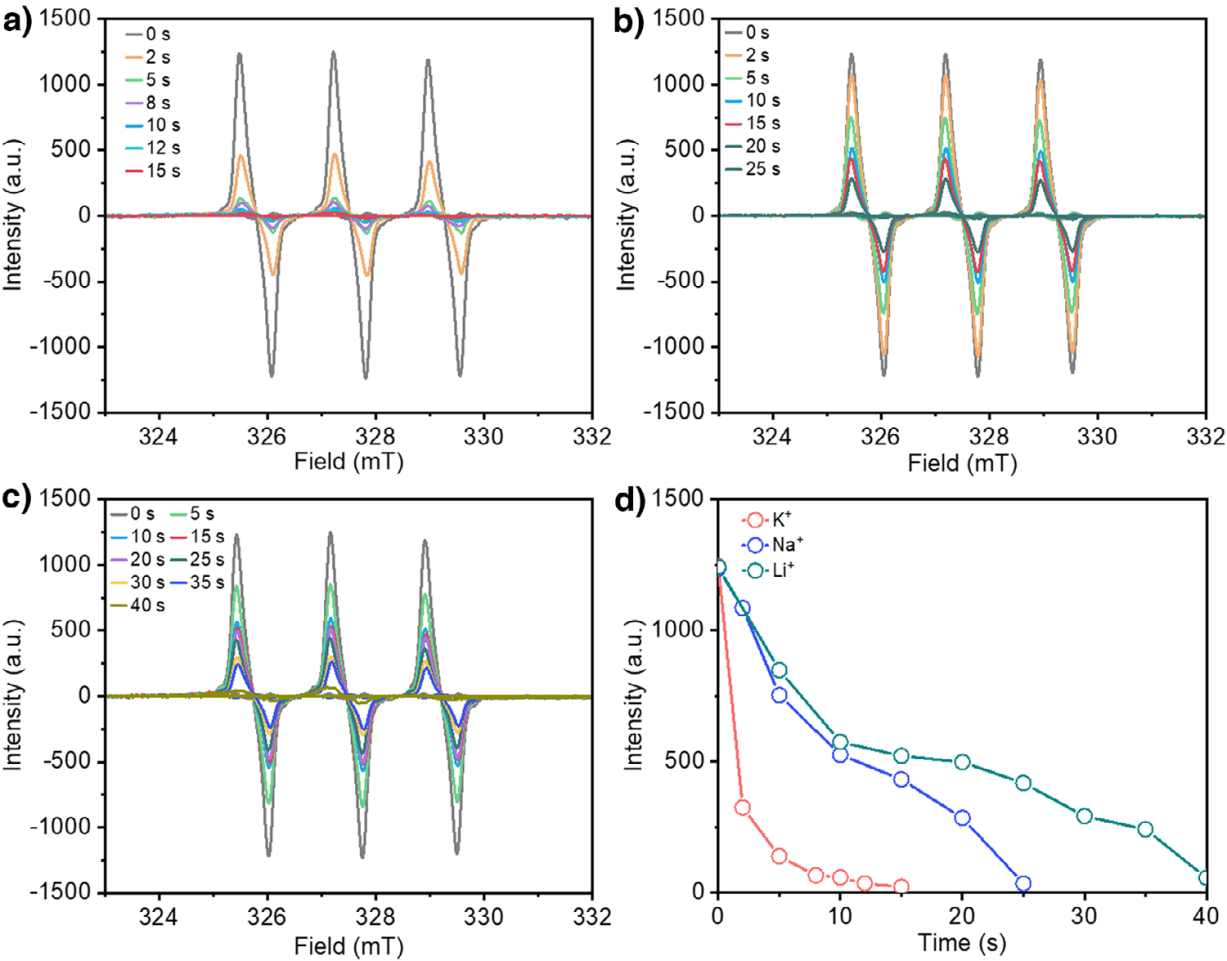
EPR experiments with TEMPO trapping using different cations in the electrolyte. EPR signal at different time intervals with IrO*_x_* at a current density of 100 mA cm^−2^ in electrolyte containing: a) K^+^, b) Na^+^, and c) Li^+^. In all cases, 0.1 mM TEMPO was added to the electrolyte. d) Corresponding change of EPR signal intensity at different time intervals for each of the cases.

Intrigued by these findings, we performed in situ Raman spectroscopy experiments to detect *Cl adsorbates on the IrO*_x_* surface under operating conditions. For all three alkali metal cations, we observed three peaks at around 172, 325, and 348 cm^−1^, which are characteristic features of Ir–Cl bonding.^[^ ^34^, ^35^ ^]^ These Ir–Cl Raman peaks were first observed at 0.9 V versus Ag/AgCl with K^+^ or Na^+^ (Figures 4a,b and S15a), while these appear later at 1.0 V versus Ag/AgCl with Li^+^ (Figures 4c and S15b). We note that this matches well with the LSV results (Figure 2a) where lower overpotentials were observed with K^+^ and Na^+^ as compared to Li^+^.

**Figure 4:**
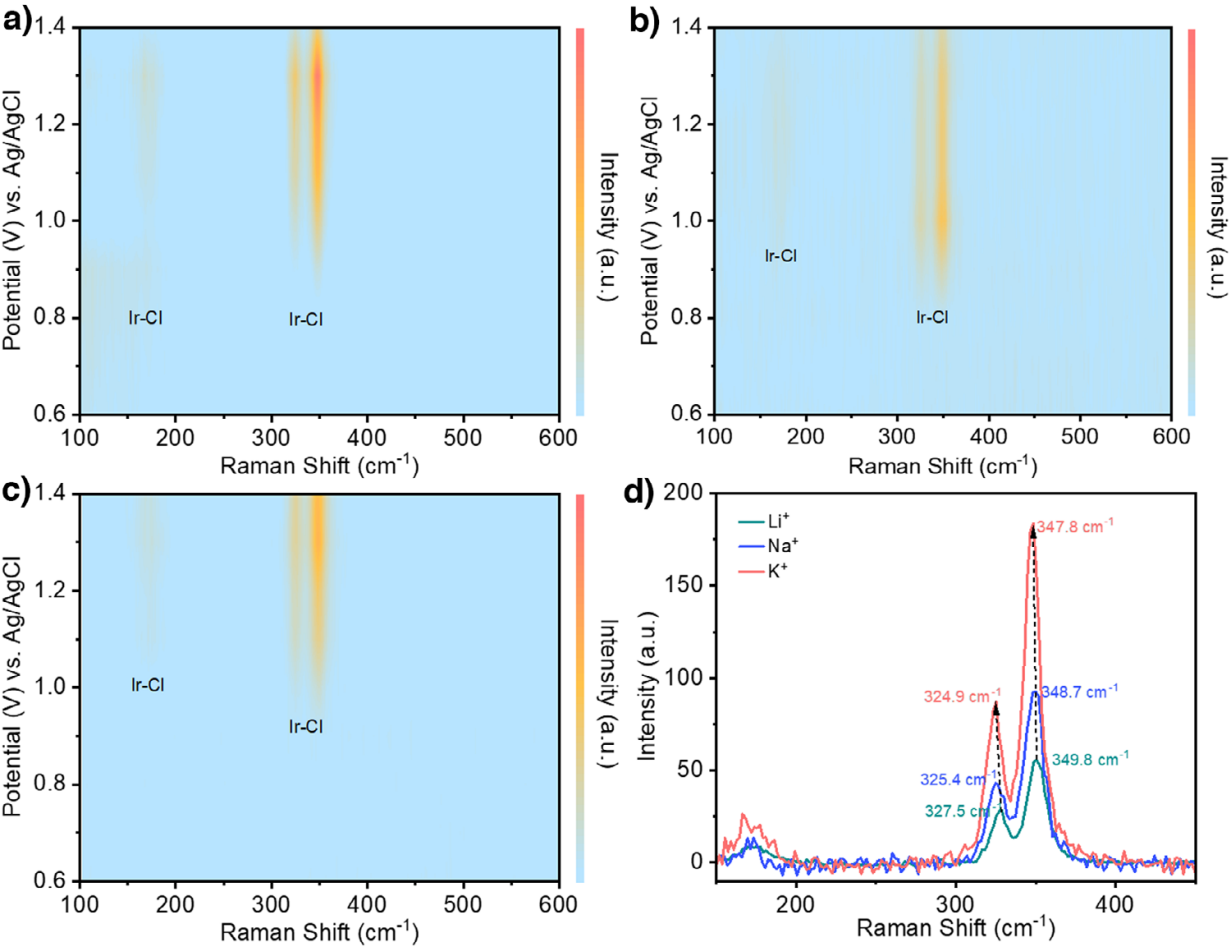
In situ Raman spectroscopy experiments. Results under different applied potentials using electrolyte containing: a) K^+^, b) Na^+^, and c) Li^+^. d) Data collected at a potential of 1.3 V versus Ag/AgCl using electrolytes containing various alkali metal cations.

The in situ Raman spectroscopy heatmaps (Figure 4a–c) also display variations in the peak intensities as the applied potential is changed. For Na^+^ and Li^+^ electrolytes, the peak intensities continually increase with a higher anodic potential (Figure 4b–c). However, for the K^+^ electrolyte these peaks have a maximum intensity at 1.3 V versus Ag/AgCl and thereafter decrease at the higher anodic potentials (Figure 4a). We postulate that this decrease in intensity could be due to a lowered *Cl coverage on the catalyst surface due to an increased propensity for Cl free radical formation. This is consistent with the K^+^ electrolyte yielding a higher chlorocyclohexane FE as compared to the Na^+^ and Li^+^ electrolytes (Figure 2d).

Importantly, we also find a trend in the peak positions from K^+^ to Li^+^. For instance, at 1.30 V versus Ag/AgCl the peaks in the K^+^ electrolyte appear at 324.9 and 347.8 cm^−1^, while these same peaks are observed in Na^+^ are at 325.4 and 348.7 cm^−1^ and Li^+^ at 327.5 and 349.8 cm^−1^ (Figure 4d). These shifts in peak position suggest a weakened Ir─Cl bond and hence a lower *Cl binding energy on the IrO*_x_* surface with a larger cation size. These results are supported by DFT calculations (Figures 5a–c and S16–S18), where we found that the *Cl binding energy is the lowest in the presence of K^+^. Specifically, this has values of −1.12 eV with K^+^, −1.25 eV with Na^+^, and −1.49 eV with Li^+^ (Figure 5d). Vibrational frequency calculations also show that the stretching vibration (z direction) indeed moves toward a higher wavenumber following a trend of K^+^ < Na^+^ < Li^+^ (Figure S19 and Table S5), as a result of a weaker *Cl binding energy with larger cation size. Additionally, we found that the variation in *Cl binding energy correlates well with the exponential relationship of cation acidity^[^ ^36^ ^]^ (Figure S20).

**Figure 5:**
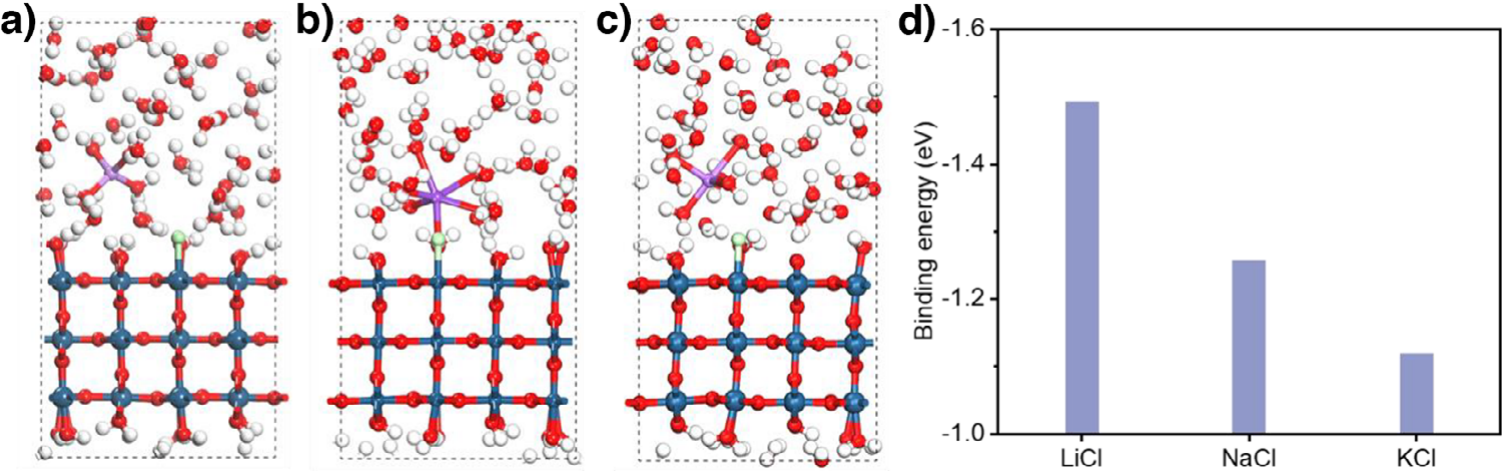
DFT calculations of the *Cl binding energy with various cations. Side views of *Cl adsorption on the IrO_2_ slab model in the presence of: a) Li^+^, b) Na^+^, and c) K^+^. White, red, green, purple, and blue spheres represent H, O, Cl, Li/Na/K, and Ir, respectively. d) Comparison of *Cl binding energies calculated using an explicit water environment in the presence of either Li⁺, Na⁺, or K⁺.

Overall, we posit that the lower *Cl binding energy could explain why there is a higher propensity for Cl radical formation and hence a higher selectivity toward chlorocyclohexane formation with the K^+^ electrolyte. These findings are depicted in the form of a schematic in Figure 6. To understand the origin of the observed differences in *Cl binding energy among the various alkali metal cations, we also conducted electron density difference calculations. The results show a more pronounced electron density redistribution in the presence of K^+^, with a resulting weaker binding energy (Figure S21). Furthermore, prior literature proposed that “cation effects” arise due to their impact on the interfacial electric field,^[^ ^29^, ^37^ ^]^ which in turn influences adsorbate binding energies. From our calculations, we found that the binding energy of *Cl on IrO_2_ does indeed vary considerably with the interfacial electric field strength (Figure S22). Hence, this could also explain why alkali metal cations affect the *Cl binding energy in our work. Finally, the impact of electrolyte cations on the adsorption of Cl^−^ (chloride anions) could also be another important factor for consideration in future studies.

**Figure 6:**
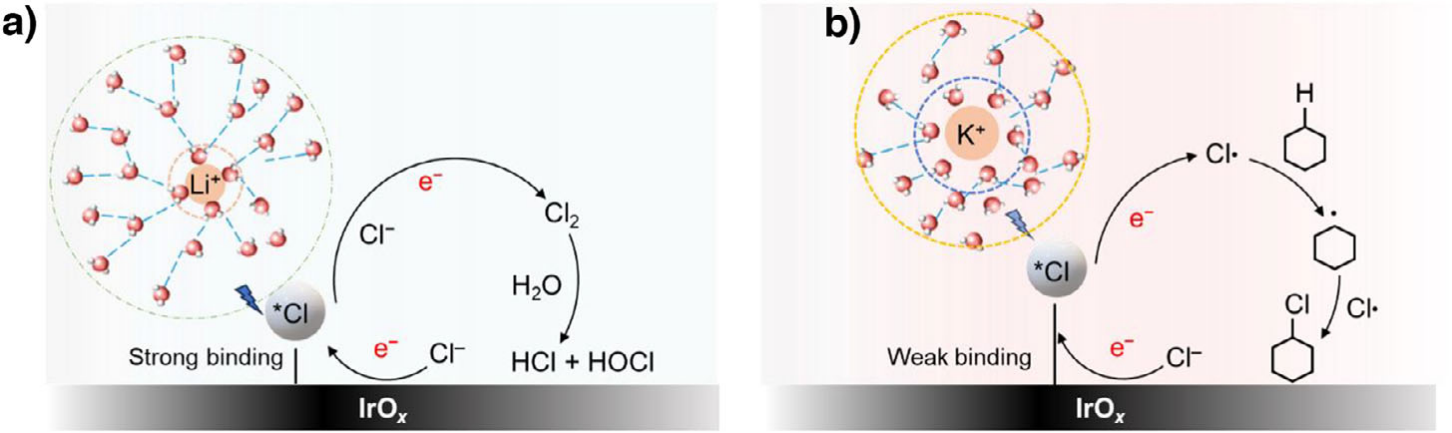
Effect of alkali metal cation on cyclohexane chlorination. Illustration of the influence of Li^+^ (a) and K^+^ (b) on the *Cl binding energy. With Li^+^, the *Cl binding energy is stronger, which results in an increased probability for Cl_2_ formation. As for K^+^, the *Cl binding energy is weaker, which favors the formation of Cl radicals needed for the chlorination of cyclohexane.

In conclusion, for this work we investigated the impact of alkali metal cations in the electrolyte on the electrochemical C─H chlorination of cyclohexane to chlorocyclohexane. Using IrO*_x_* as the working electrode, we found that the FE toward chlorocyclohexane trends according to K^+ ^> Na^+ ^> Li^+^. Specifically, a 90.3% FE was obtained with 1 M KCl electrolyte as compared to 78.4% with 1 M LiCl electrolyte for the conversion of cyclohexane to chlorocyclohexane at 1000 mA. This “cation effect” was studied using EPR spectroscopy with TEMPO trapping, where we found evidence of an increased propensity for the generation of Cl radicals when larger cations are present. DFT calculations and in situ Raman spectroscopy both indicate that this could be due to a decrease in the *Cl binding energy of IrO*_x_* with K^+^ electrolyte. Our results offer insights into how the reaction environment can be tuned to enhance electrochemical organic transformations.

## Author Contributions

Y.L. supervised the project. Y.L. and B.Wu. conceived the idea and designed the experiments. B.Wu. and B.C carried out all the experimental work. B.Wu and B.Wang. carried out the catalyst characterization. T.Y. and B.Z. carried out the in situ Raman spectroscopy experiments. S.X. conducted the XAS experiments. R.L. and Z.W. performed and supervised the computational work respectively. B.Wu. and Y.L. co‐wrote the manuscript. All authors discussed the results and assisted during the manuscript preparation.

## Conflict of Interests

The authors declare no conflict of interest.

## Supporting information



Supporting Information

## Data Availability

The data that support the findings of this study are available from the corresponding author upon reasonable request.
