# Supplementary material for: Alkali Metal Cations Impact the Selectivity of Radical‐Mediated Electrochemical C─H Chlorination
Source: Angew Chem Int Ed Engl. 2025 Jul 23;64(36):e202509115. doi: 10.1002/anie.202509115 (PMC12402841; doi:10.1002/anie.202509115)
Supplement: Supplementary file 1 — Supporting Information [file ANIE-64-e202509115-s001.pdf]

Supporting Information

Alkali Metal Cations Impact the Selectivity of Radical-Mediated Electrochemical C-H Chlorination

Bo Wu[1,2,6], Ruihu Lu[3,6], Tenghui Yuan[4,6], Beijing Cai[1], Bingqing Wang[1], Bote Zhao[4], Shibo Xi[5], Ziyun Wang[3]* and Yanwei Lum[1,2]*

[1]Department of Chemical and Biomolecular Engineering, National University of Singapore, Singapore, 117580, Republic of Singapore.

[2]Institute of Materials Research and Engineering (IMRE), Agency for Science, Technology and Research (A*STAR), 2 Fusionopolis Way, Innovis #08-03, Singapore 138634, Republic of Singapore.

[3]School of Chemical Sciences, The University of Auckland, Auckland, 1142, New Zealand.

[4]School of Environment and Energy, South China University of Technology, Guangzhou, 510006, China.

[5]Institute of Sustainability for Chemical, Energy and Environment (ISCE[2]), Agency for Science, Technology and Research (A*STAR), 1 Pesek Road, 627833, Republic of Singapore.

[6]These authors contributed equally to this work.

*Corresponding author: ziyun.wang@auckland.ac.nz

*Corresponding author: lumyw@nus.edu.sg

Methods

Materials. Lithium sulfate (99.5%), sodium sulfate (ACS regent, ≥99%), potassium sulfate (ACS regent, ≥99%), hydrochloric acid (ACS reagent, 37%), cyclohexane (>99.0%), oxalic acid (≥98%), 2,2,6,6-tetramethylpiperidine oxide (TEMPO), were purchased from Sigma-Aldrich. Iridium(III) chloride (99.8%) was purchased from Alfa Aesar. Isopropanol (ACS reagent, 99.8%) was purchased from Fisher Chemicals. Sulfuric acid (98%) was obtained from J.T.Baker. Deionized (DI) water (18.2 MΩ) was obtained using an OmniaPure UltraPure Water System (Stakpure GmbH), which was used for all the experiments. Nafion exchange membrane (Nafion 117, size of 2.5 cm by 2.5 cm, with a thickness of 0.18 mm) and Ti screen mesh were purchased from Fuel Cell Store. The electrochemical H-type cell and Ag/AgCl (3M KCl) reference electrodes were purchased from Tianjin Aida Hengsheng Technology Development Co., Ltd. All the chemicals used in this work were of analytical grade and used without further purification. The pretreatment and activation of Nafion 117 membrane was done by keeping the membrane for 1 h in 5 wt.% H_2O_2 solution under 80 °C, followed by washing with DI water. Following this, the membrane was immersed in 0.5 M H_2SO_4 for another 1 h under 80 °C and then washed again with DI water.

IrO_x catalyst preparation. IrO_x was synthesized on Ti mesh-based support through a two-step method (dip coating and thermal decomposition method), as previously described by Luc et al[1]. Ti mesh was firstly etched for 1 h in a boiling 0.5 M oxalic acid solution (80 °C). After that, the mesh was dipped coated into a 10 ml isopropanol solution with 10% volume concentrated HCl containing 30 mg of dissolved $IrCl_3 \cdot (H_2O)_x$. This process was followed by drying at air under 100 °C for 10 min and calcination at 500 °C for 10 min in air. Those procedures were repeated 10 times to achieve a catalyst loading of 1 mg/cm^2.

Electrochemical measurements. The cyclohexane chlorination experiments were conducted in a H-type cell configuration (see illustration in Fig. S4) consisting of the IrO_x based anode

catalyst, cation exchange membrane (Nafion 117), carbon rod as the cathode and Ag/AgCl (saturated KCl) as the reference electrode. 45 ml of electrolyte (0.5 M H_2SO_4 + 1 M XCl, where X represents Li, Na or K) mixed with 5 ml of cyclohexane was used for the anolyte, while 50 ml 0.5 M H_2SO_4 was used for the catholyte. Electrochemical measurements in this work were carried out using an Autolab PGSTAT204 with a current booster. The reported current densities are based on the geometric surface area of 5 cm^2 for the anode. After 30 min of electrolysis, the organic phase containing product and unreacted cyclohexane was separated from the aqueous electrolyte using a separating funnel and collected for product analysis and quantification. The quantification of the product was based on the GC-MS results (Schematic illustration of these processes had been added as Fig. S5).

Linear sweep voltammetry experiments. These tests were carried out in a three-electrode configuration cell using a carbon rod as the counter-electrode and Ag/AgCl (saturated KCl) as the reference electrode. The working electrodes were prepared by drop-casting catalyst ink onto a glassy carbon electrode (5 mm in diameter). The rotating disk electrode (RDE) system was from Pine Instrument Company, USA to control the electrode rotation speed. The scan rate was set at 10 mV/s from 0.5 to 1.8 V (versus Ag/AgCl) in different electrolyte using a potentiostat system (PARSTAT MC, Princeton Applied Research, US). The IR correction (85%) was conducted based on the solution resistance obtained from electrochemical impedance spectroscopy (EIS) measurements.

Product analysis and quantification. A combination of Fourier-transform infrared spectroscopy (FTIR), and gas chromatography mass spectrometry (GCMS) were used to analyse and quantify the products. FTIR was carried out on a Bruker Vertex 70 system equipped with a MIR-FIR ATR. The background (8 cm^{-1} resolution, 32 scans) was collected under ambient environmental conditions. After the baseline subtraction, the sample signal was collected after 32 scans.

Quantification of the products was carried out using GCMS on an Agilent 5977C GC/MSD equipped with a J&W HP-5ms capillary column (Part Number:19091S-433). Vials containing the liquid samples were placed into an autosampler holder, and 0.2 μL of sample was injected into the column with a split mode of 1:500. The GC used a temperature program during which the temperature was held at 50 °C for 5 min. Following this, the temperature was increased to 250 °C with a ramp rate of 15 °C/min. The temperature was then maintained at 250 °C for a further 7 min. All reported Faradaic efficiencies (FE) in this work were averaged from at least three different independent electrolysis experiments. The FE towards chlorocyclohexane was calculated according to the following equation:

$$\text{Faradaic efficiency (FE)} = \frac{F \cdot n_a \cdot V_{organic} \cdot c_{product}}{t \cdot i_{overall}}$$

Where F is the Faraday constant, n_a (a value of 2 for this work) is the number of the electron transfers required for 1 mol chlorocyclohexane production. $V_{organic}$ stands for the volume of the organic solution, $c_{product}$ is the concentration of the product in the organic solution, t is electrolysis duration (30 min in this work) and $i_{overall}$ is the applied current.

Materials characterization. The morphologies of the electrodes were investigated using scanning electron microscopy (SEM) with a JSM-7610FPlus (JEOL) at a 5 kV beam voltage. Transmission electron microscopy (TEM) was conducted on a Hitachi HF-3300 equipped with a Bruker energy dispersive x-ray spectroscopy detector at an acceleration voltage of 300 kV. Ir L_3-edge X-ray absorption spectroscopy measurements were performed at the XAFCA beamline of Singapore Synchrotron Light Source (SSLS) under transmission mode.

***In-situ* Raman spectroscopy**. *In-situ* Raman measurements were carried out using a LabRAM HR 800 Raman spectrometer with a custom electrochemical cell. Spectra were collected using an objective lens (×50) and a 532 nm laser (10% intensity). Pt wire and Ag/AgCl (KCl saturated) were used as the counter and reference electrodes respectively. IrO_x synthesized onto a Au substrate through the same thermal decomposition method. For these experiments, different

electrolytes containing different cations were used as the electrolyte. *In-situ* Raman spectra was then collected at various constant potentials (from 0.6 V to 1.4 V vs. Ag/AgCl) for 15 consecutive scans and 10 s exposure time per scan.

Electron paramagnetic resonance (EPR) testing. The EPR testing was conducted on a JEOL FA200 ESR. TEMPO was mixed with the KCl electrolyte and cyclohexane for EPR testing. A current of 400 mA was applied using IrO_x as the anode catalyst for different anolyte containing different metal cation mixed with cyclohexane under various reaction durations, in which 0.1 mM TEMPO was used for all cases. Samples were collected and analyzed quickly using EPR (within 30 s). A reduced TEMPO signals was observed during the reaction

Iodometric titration. Iodometric titration of the anolyte was conducted by first adding an excess of 10% KI solution to react with the unreacted chlorine/hypochlorite species to form iodine. This was followed by addition of starch solution to form a dark blue starch-iodine complex. Next, this was then titrated with 1 M NaS_2O_3 solution until the solution turned clear again, and the amount of NaS_2O_3 was recorded and used to determine the FE of unreacted chlorine/hypochlorite species.

DFT calculations. Density functional theory (DFT) calculations were performed by using ab initio simulation package (VASP)[2-4]. The generalized gradient approximation (GGA) in the Perdew-Burke-Ernzerhof functional was adopted to describe the electron exchange and correlation energy[5], and the frozen-core projector-augmented wave method with a cutoff energy of 400 eV was chosen to describe the interaction between core electrons and valence electrons[6]. The long-range vdw interactions between atoms are finely described by the DFT-D3 correction method in Grimme's scheme[6]. The criteria of energy and force convergence are set to 1.0×10^{-5} eV per atom and 0.02 eV·Å^{-1}, respectively, for geometry optimization. And a Γ-centred Monkhorst–Pack k-point mesh grid of 1×1×1 was employed for all structural optimizations[7].

To simulate the effect of alkaline metal ions, such as Li^+, we adopted the explicit water environment on IrO_2 surface. We first constructed three-layer (3 × 2) IrO_2(110) slab to represent IrO_2 surface, including 36 Ir and 72 O atoms. And the densities of bulk water in this model were maintained close to 1g/cm^3, including 55 H_2O molecules and one metal atom and Cl atom within a lattice of 9.56 × 12.85 Å^2. In the model, we built adsorbed state or not adsorbed state for Cl atom to calculate the adsorption energy. To obtain a well-equilibrated water structure, we used ab initio molecular dynamics (AIMD) simulations[8], we conducted Canonical ensemble (NVT) simulations with a fixed number of atoms (N), a fixed volume (V), and a fixed temperature (T) were performed with a timestep of 0.2 fs, using the Nose–Hoover thermostat at a temperature of 298 K with a damping parameter of 10 fs. After achieving a stable water structure, we further replace the metal ion and optimized water structure through optimization.

We considered valence electrons-H (ultrasoft test, $1s^1$), Li($1s^2$, $2s^1$, $2p^0$), O($2s^2$, $2p^4$), Na($2p^6$, $3s^1$), Cl($3s^2$, $3p^5$), K($3s^2$, $3p^6$, $4s^1$), and Ir($5d^8$, $6s^1$) in our calculations with solvated IrO_2 surface. The projector-augmented wave (PAW) pseudopotentials were used as implemented in the VASP 5.4 code to treat core–electron interactions. Partial occupancies were determined using the Gaussian smearing method with a width of 0.2 eV (ISMEAR = 0). Structural optimizations were performed using the conjugate gradient algorithm (IBRION = 1) with a step width of 0.2 Å. Electronic minimization was performed using the blocked Davidson algorithm (ALGO = Fast).

The adsorption energy of *Cl is calculated listed:

$$\Delta E_{\mathrm{ads}} = E_{*\mathrm{Cl}} - E_{*/\mathrm{Cl}}$$

where E_{*Cl} and $E_{*/Cl}$ represent the energy of solvated IrO_2 surface with *Cl adsorption and solvated Cl ion, respectively. The more negative ΔE_{ads} value, the stronger the adsorption energy.

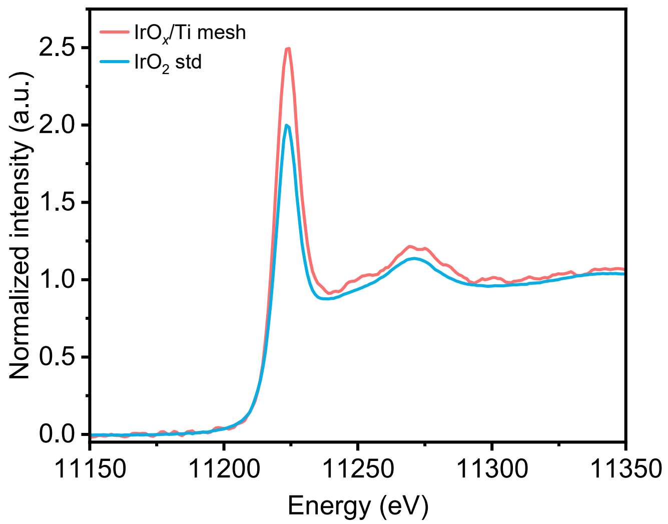

Fig. S1. Ir L_3-edge XANES spectra of the as prepared IrO_x/Ti mesh and IrO_2 standard.

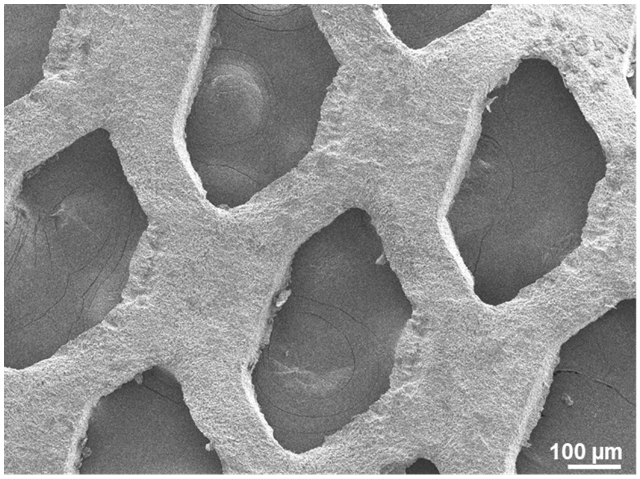

Fig. S2. SEM image of the as prepared IrO_x/Ti mesh.

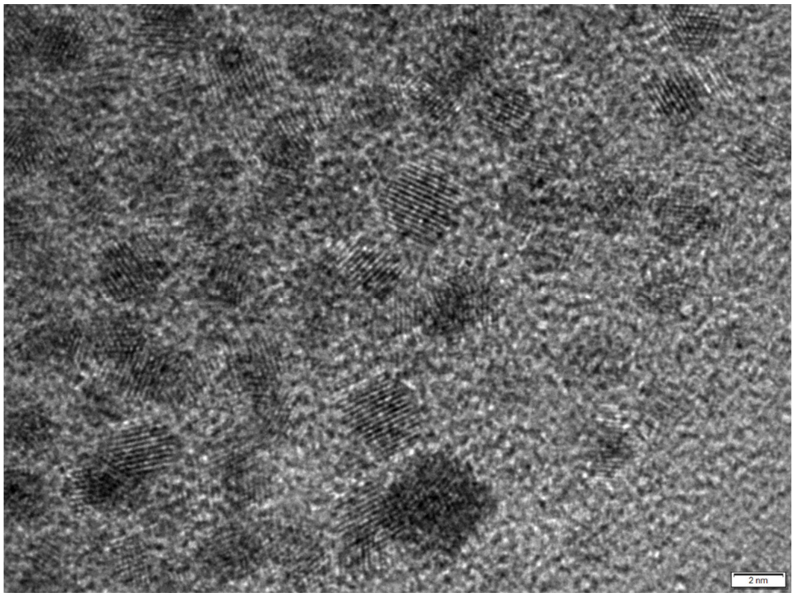

Fig. S3. TEM images of the IrO_x particles, with scale bar width representing 2 nm.

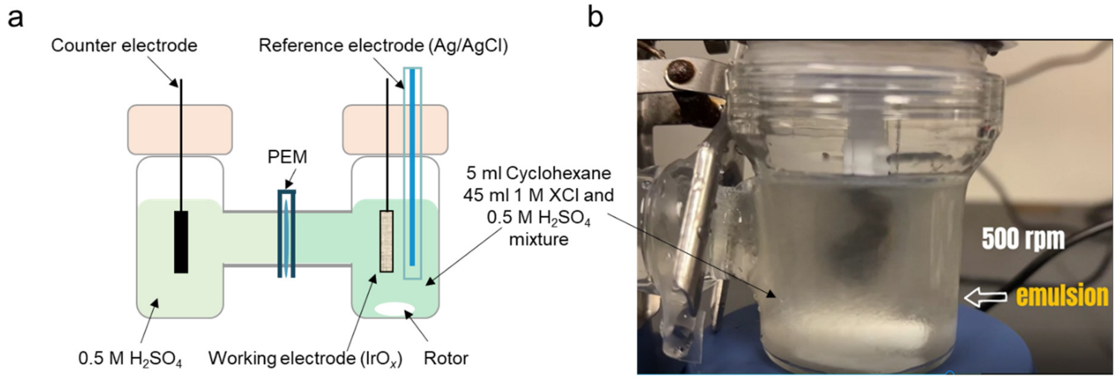

Fig. S4. (a) Schematic illustration of chlorocyclohexane production in the H-type cell. Here XCl in the illustration represents KCl, NaCl, or LiCl. (b) Picture of H-type cell during the reaction. To ensure good mixing of the emulsion, a high rotation speed (500 rpm) was used to maintain rapid agitation.

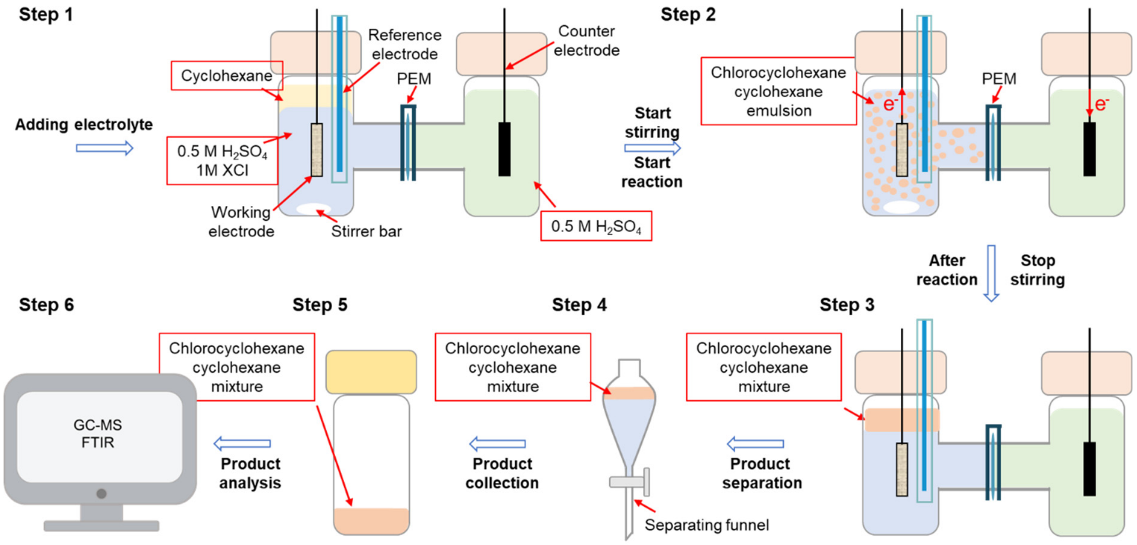

Fig. S5. Schematic illustration of the process for the conversion of cyclohexane to chlorocyclohexane. XCl represents KCl, NaCl, or LiCl.

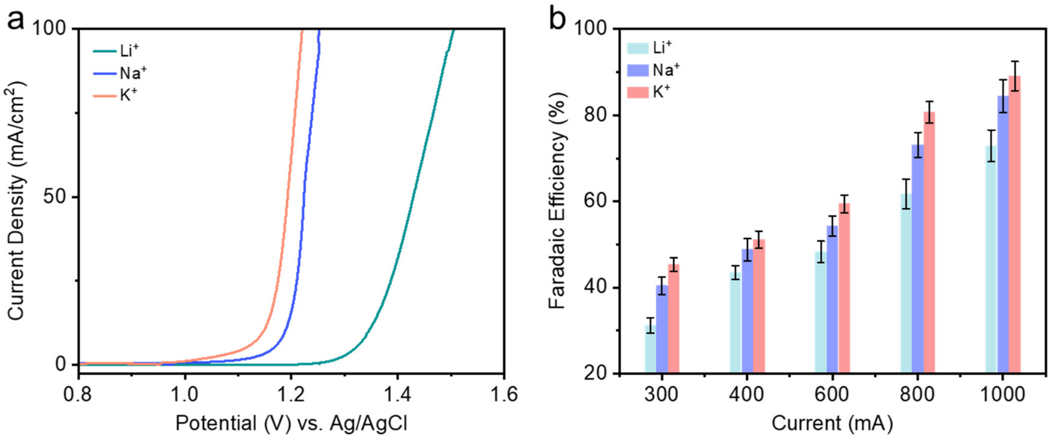

Fig. S6. LSV curves with 85% IR (voltage drop) correction with various 1 M XCl, where X represents either Li, Na or K. In this case, sulfuric acid was not added as the supporting electrolyte. 5 ml of cyclohexane was added to 45 ml of electrolyte and rapidly stirred to maintain an 'emulsion'-like state. (b) Chlorocyclohexane FE at different applied currents for the various 1 M XCl solution, where X represents either Li, Na or K. Note: quantification was based on GCMS analysis and the geometric area of electrode used in all cases was 5 cm^2.

Table S1 Electrochemical potential under various constant applied currents in the various cation electrolytes. Note: the geometric area of electrode used in all cases was 5 cm^2.

Current (mA)	Potential (V) vs. Ag/AgCl in 1 M KCl	Potential (V) vs. Ag/AgCl in 1 M NaCl	Potential (V) vs. Ag/AgCl in 1 M LiCl
300	1.23	1.25	1.27
400	1.25	1.27	1.29
600	1.26	1.30	1.31
800	1.28	1.33	1.35
1000	1.30	1.35	1.38
1200	1.31	1.37	1.40
1300	1.32	1.38	1.41

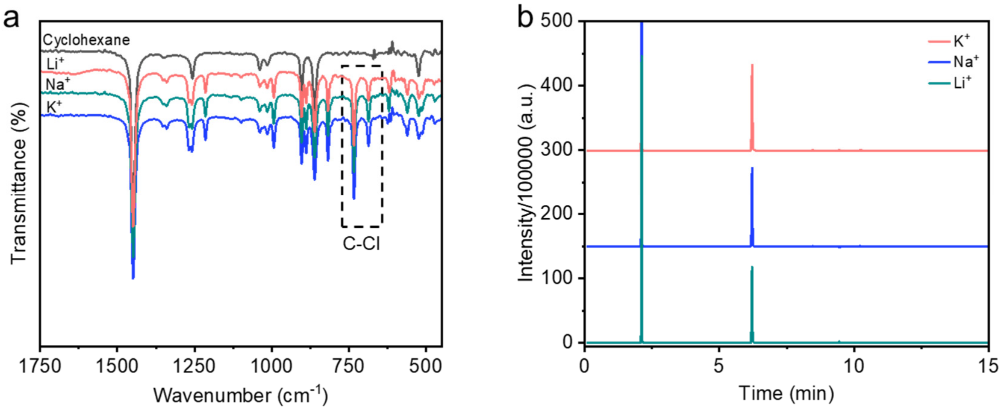

Fig. S7. Comparison of the (a) FTIR spectroscopy and (b) GCMS results obtained in electrolytes containing different alkali metal cations at 1000 mA.

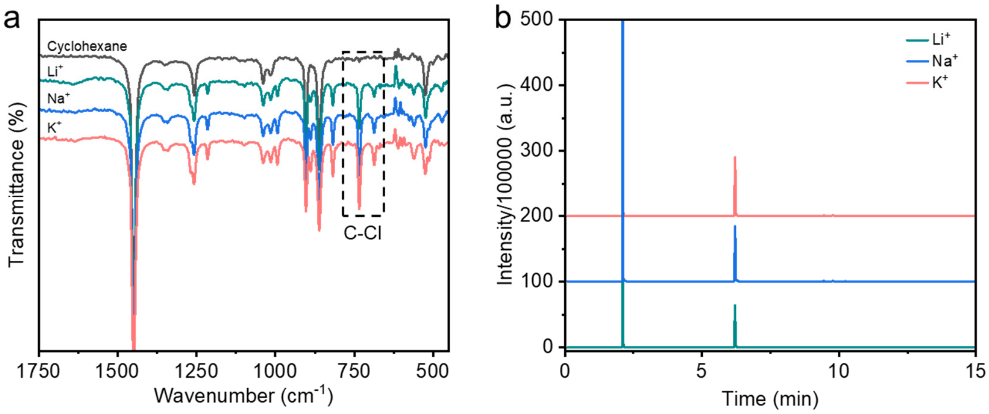

Fig. S8. Comparison of the (a) FTIR spectroscopy and (b) GCMS results obtained in electrolytes containing different alkali metal cations at 800 mA.

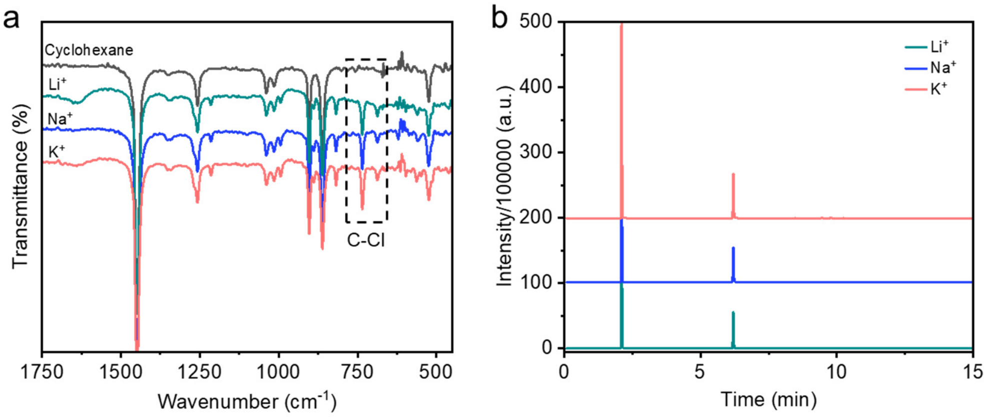

Fig. S9. Comparison of the (a) FTIR spectroscopy and (b) GCMS results obtained in electrolytes containing different alkali metal cations at 600 mA.

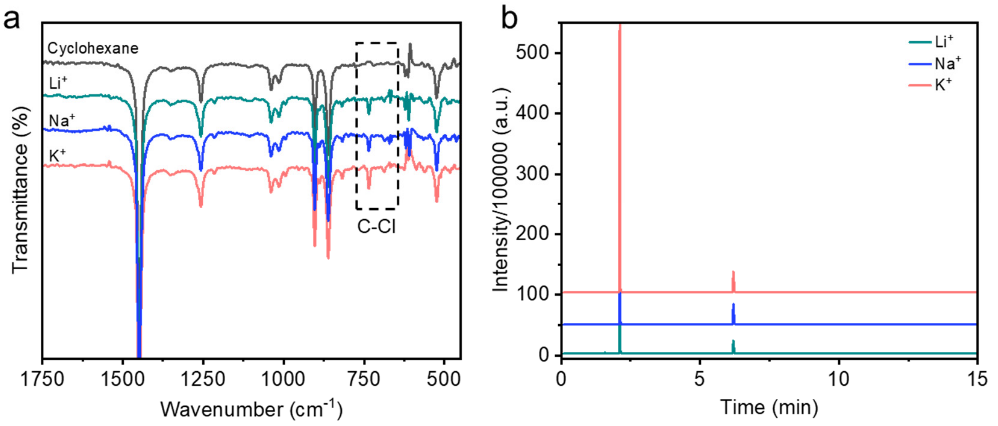

Fig. S10. Comparison of the (a) FTIR spectroscopy and (b) GCMS results obtained in electrolytes containing different alkali metal cations at 400 mA.

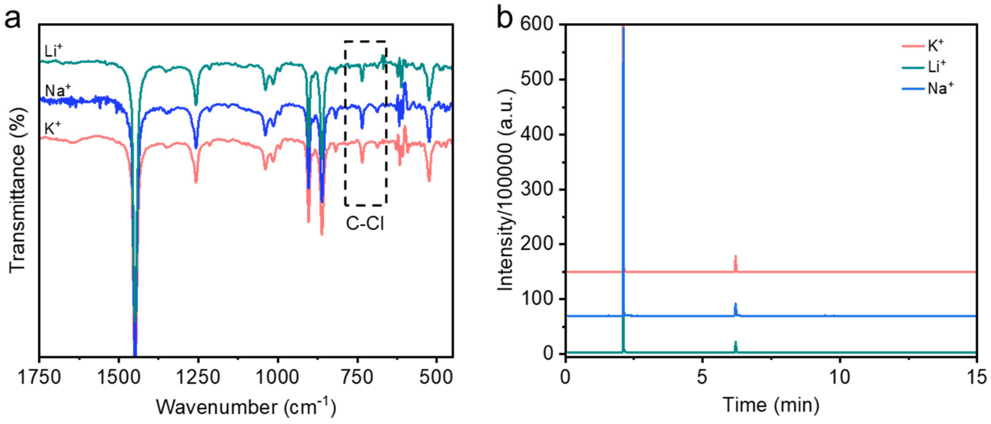

Fig. S11. Comparison of the (a) FTIR spectroscopy and (b) GCMS results obtained in electrolytes containing different alkali metal cations at 300 mA.

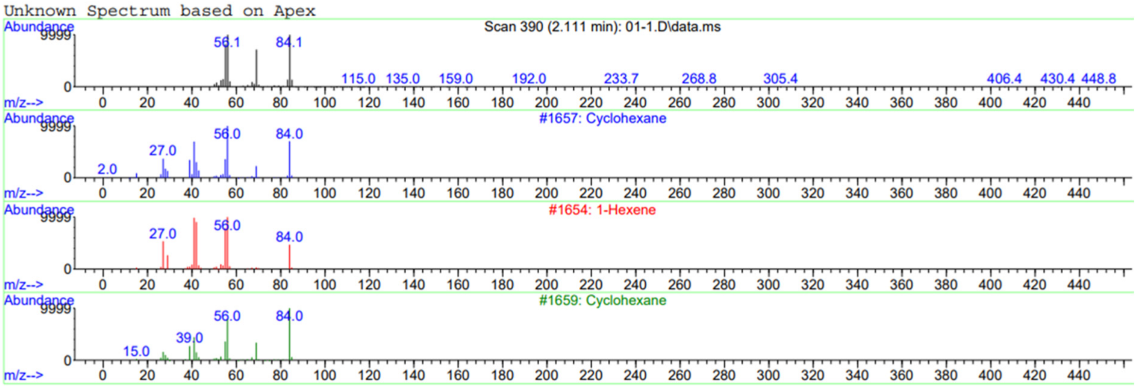


```
Data File: S:\gcms\External\ChBE\20240304_Lum\01-1.D
Sample    : 1

Peak Number:  1     at      2.111 min   Area: 817425604

 The 5 best hits from each library.            Ref#  CAS#              Qual MW. Formula
-----------------------------------------------------------------------------------------
    C:\Database\NIST23.L
     1 Cyclohexane                              1657 000110-82-7       72    84 C6H12
```

Fig. S12. MS spectrum of our sample from the GCMS peak at around 2.1 min, which corresponds to the unreacted cyclohexane.

Unknown Spectrum based on Apex

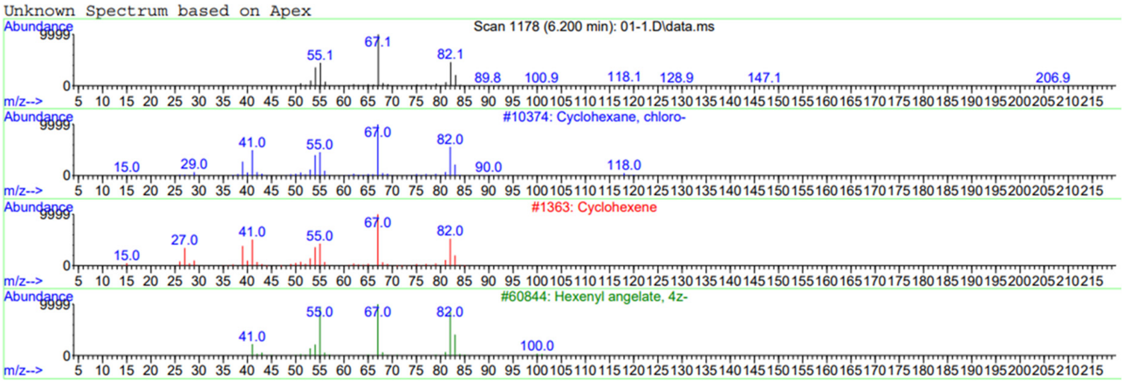


```
Data File: S:\gcms\External\ChBE\20240304_Lum\01-1.D
Sample   : 1

Peak Number:  2     at      6.200 min  Area:  61859093

 The 5 best hits from each library.           Ref#  CAS#             Qual MW. Formula
-----------------------------------------------------------------------------------------
    C:\Database\NIST23.L
     1 Cyclohexane, chloro-                    10374 000542-18-7      83   118 C6H11Cl
```

Fig. S13. MS spectrum of our sample from the GCMS peak at around 6.2 min, which corresponds to the chlorocyclohexane product.

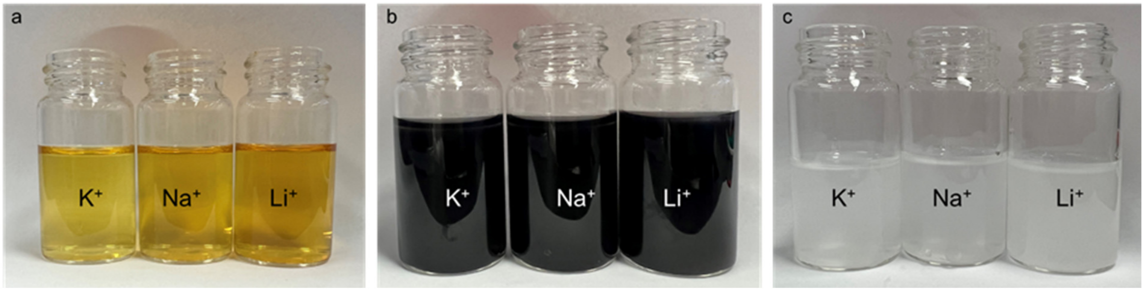

Fig. S14. (a) Digital photograph of the anolyte after addition of excess 10% KI solution with different metal cation in the electrolyte. A brown coloration is observed due to oxidation of I^- to form I_2. (b) Digital photograph of the same anolyte after starch solution was added, forming a dark blue starch-iodine complex. (c) Digital photograph of the anolyte after titration with $Na_2S_2O_3$, yielding a clear colourless solution.

The equation for the reaction between I_2 and $S_2O_3^{2-}$ is shown below:

$$2S_2O_3^{2-} + I_2 \rightarrow S_4O_6^{2-} + 2I^-$$

Table S2. FE towards unreacted chlorine/hypochlorite species at various applied currents for the K^+ electrolyte.

Current	Amount of $Na_2S_2O_3$ added (mmol)	Amount of unreacted chlorine/hypochlorite species	FE loss due to the unreacted hypochlorite (%)
300	2.4	1.2	42.7%
400	2.3	1.2	30.1%
600	2.0	1	18.2%
800	1.8	0.9	12.0%
1000	1.7	0.85	8.8%
1200	1.9	1	8.5%
1300	5.6	2.8	25%

Table S3. FE towards unreacted chlorine/hypochlorite species at various applied currents for the Na^+ electrolyte.

Current	Amount of $Na_2S_2O_3$ added (mmol)	Amount of unreacted chlorine/hypochlorite species	FE loss due to the unreacted hypochlorite (%)
300	2.7	1.35	48.1%
400	3.4	1.7	43.8%
600	3.1	1.55	26.5%
800	2.6	1.3	17.1%
1000	2.4	1.2	12.1%
1200	3.6	1.8	15.9%
1300	8.2	4.1	36.4%

Table S4. FE towards unreacted chlorine/hypochlorite species at various applied currents for the Li^+ electrolyte.

Current	Amount of $Na_2S_2O_3$ added (mmol)	Amount of unreacted chlorine/hypochlorite species	FE loss due to the unreacted hypochlorite (%)
300	3.3	1.6	58.8%
400	3.7	1.9	47.6%
600	4.3	2.2	36.8%
800	4.1	2.05	27%
1000	4.2	2.1	21.2%
1200	6.2	3.1	27.3%
1300	9.3	4.6	41.2%

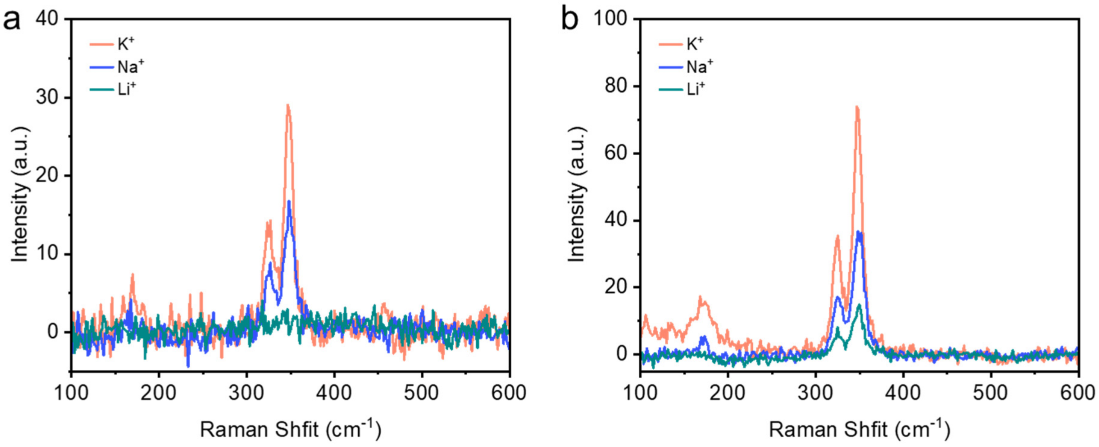

Fig. S15. *In-situ* Raman spectroscopy data collected at an applied potential of (a) 0.9 V and (b) 1.0 V vs. Ag/AgCl using electrolytes containing either K^+, Na^+ or Li^+.

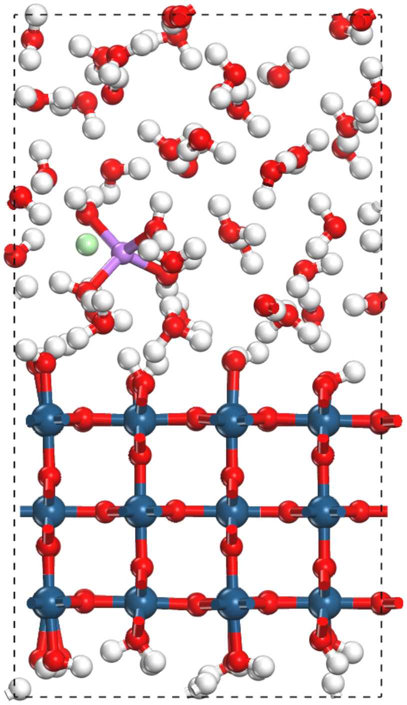

Fig. S16. Side view of Cl^- in the explicit aqueous environment. White, red, green, purple and blue spheres represent H, O, Cl, Li and Ir, respectively.

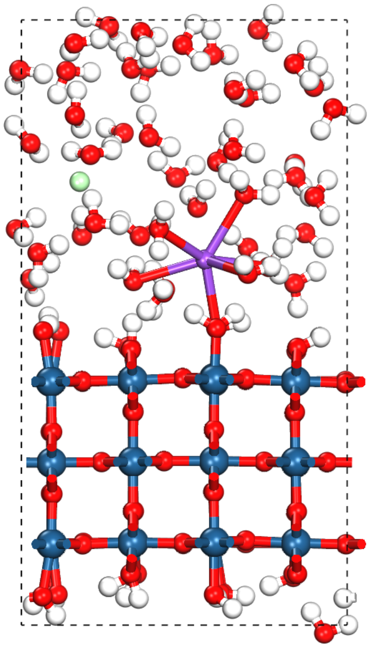

Fig. S17. Side views of Cl^{-} in the explicit aqueous environment. White, red, green, purple and blue spheres represent H, O, Cl, Na and Ir, respectively.

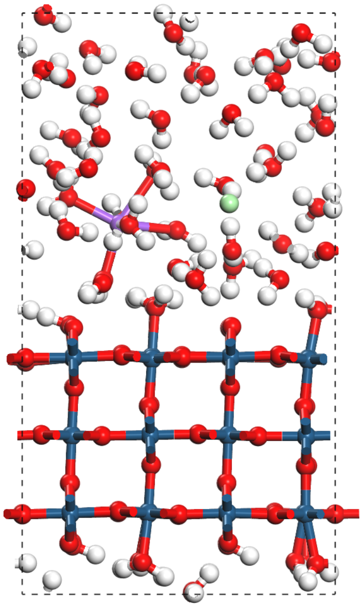

Fig. S18. Side views of Cl^- in the explicit aqueous environment. White, red, green, purple and blue spheres represent H, O, Cl, K and Ir, respectively.

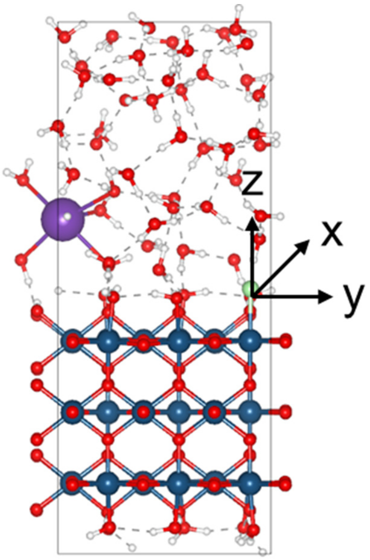

Fig. S19. Depiction of *Cl adsorbed onto the IrO_2 surface, with the x, y and z directions shown. The white, red, green, blue, and purple spheres represent H, O, Ir, Cl and alkali metal cation, respectively.

Table S5. Vibrational wavenumber of *Cl adsorbed onto the IrO_2 surface in the presence of varous alkali metal cations. The vibration directions (x, y and z) are depicted in Fig. S19.

	Wavenumber (cm^{-1})		
	1f (z direction; stretching vibration)	2f (x direction; in-plane translational vibration)	3f (y direction; in-plane translational vibration)
Li^+	304.03	115.12	121.03
Na^+	297.90	172.94	112.37
K^+	294.57	163.71	114.45

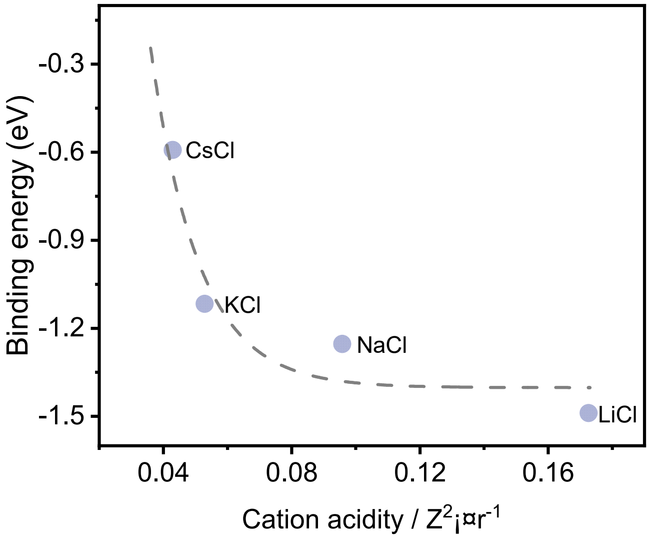

Fig. S20. Correlation between cation acidity and *Cl binding energy on the IrO_2 surface with various alkali cations.

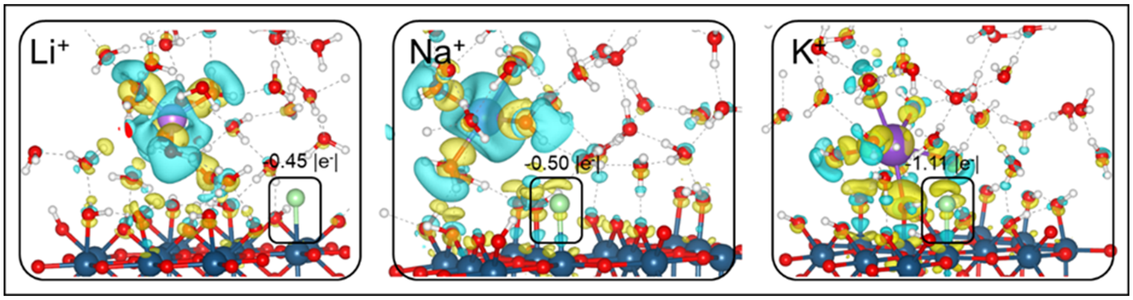

Fig. S21. Electron density difference maps of the water-Cl-IrO_2 interface after introducing Li^+, Na^+ or K^+, plotting with an isovalue of 0.0015 electrons Å^{-3}. Yellow and cyan isosurfaces represent spatial regions experiencing electron accumulation and depletion, respectively. The black box highlights the *Cl atom, with the Bader charge labelled.

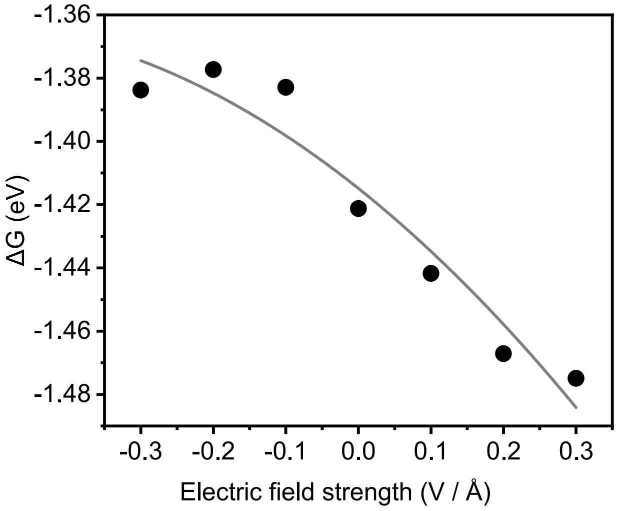

Fig. S22. Binding energy of *Cl as a function of the interfacial electric field strength. The relationship varies according to $G = G_0 + \mu\mathcal{E} - \frac{\alpha\mathcal{E}^2}{2}$. Here G refers to the binding energy, G_0 refers to the binding energy without any electric field strength, $\mathcal{E}$ refers to the electric field strength, μ refers to the adsorbate dipole moment and α is the adsorbate polarizability.

References

1 Luc, W., Rosen, J. & Jiao, F. An Ir-based anode for a practical CO_2 electrolyzer. *Catalysis Today* **288**, 79-84, doi:10.1016/j.cattod.2016.06.011 (2017).

2 Kresse, G. & Furthmüller, J. Efficiency of ab-initio total energy calculations for metals and semiconductors using a plane-wave basis set. *Computational materials science* **6**, 15-50 (1996).

3 Kresse, G. & Hafner, J. Ab initio molecular dynamics for liquid metals. *Physical review B* **47**, 558 (1993).

4 Kresse, G. & Hafner, J. Ab initio molecular-dynamics simulation of the liquid-metal–amorphous-semiconductor transition in germanium. *Physical Review B* **49**, 14251 (1994).

5 Perdew, J. P., Burke, K. & Ernzerhof, M. Generalized gradient approximation made simple. *Physical review letters* **77**, 3865 (1996).

6 Blöchl, P. E. Projector augmented-wave method. *Physical review B* **50**, 17953 (1994).

7 Monkhorst, H. J. & Pack, J. D. Special points for Brillouin-zone integrations. *Physical review B* **13**, 5188 (1976).

8 Hutchison, P., Rice, P. S., Warburton, R. E., Raugei, S. & Hammes-Schiffer, S. Multilevel computational studies reveal the importance of axial ligand for oxygen reduction reaction on Fe–N–C materials. *Journal of the American Chemical Society* **144**, 16524-16534 (2022).
